# Methyl-violet, or Pyoktanin1Read before the Pennsylvania State Veterinary Medical Association.

**Published:** 1897-08

**Authors:** W. Horace Hoskins

**Affiliations:** Philadelphia


### METHYL-VIOLET, OR PYOKTANIN; WITH REPORTS OF OTHER VETERINARIANS.[1]

By W. Horace Hoskins, D.V.S.,
PHILADELPHIA.

Methyl-violet, or pyoktanin, according to Liebreich, is a mixture of aniline compounds. Apyorin, introduced by Pettitas as an antiseptic similar to pyoktanin, is a yellow crystalline powder slightly soluble in hot and cold water, and soluble in alcohol.

Aniline was said by Mohler and Frereichs to be devoid of poisonous action, but the experiments of Dr. Letheby and the numerous cases of poisoning which have occurred from the use of aniline in the arts demonstrate that it is a powerful toxic agent. The symptoms produced by inhalation or ingestion are great prostration, heaviness in the head, giddiness, vomiting, violent neuralgic pains, and, if the dose has been large enough, cyanosis, coma, with dilatation of the pupils, excessive perspiration, loss of reflexes and of voluntary movement, hurried, weak pulse, rapid or irregular respiration, hæmoglobinuria, a peculiar discoloration of the skin, and, if the patient survive sufficiently long, jaundice with great increase

[1] Read before the Pennsylvania State Veterinary Medical Association.

in the biliary pigment of the urine. At an autopsy report by Muller (A. J. P., 1887), in a woman killed by the swallowing of about a fluidounce (29 c.c.) of aniline, the blood was chocolate-brown and gave the spectrum of methæmoglobin, the urine was free from sugar, albumin or blood, but contained paraheidophenol, and its distillations gave aniline reactions. Its destructive power on red blood-corpuscles has been so marked that they have been known to fall from 5,000,000 per c.c. to 2,700,000 on the seventh and 1,400,000 on the eleventh day.

The violent effects of the aniline dyes locally, which have been noticed by some, have probably been due to the presence of arsenic, which is used in the production of aniline and may remain as a contamination. Methyl-blue hypodermatically given was found fatal in doses of three decigrammes per kilo in weight, with symptoms of prostration, loss of response to external irritants, anuria, and chocolate discoloration of the blood.

Janicke, experimenting with the pathognomonic organisms of pus of anthrax, of cholera, of typhus, and of pneumonia, found the activity of pyoktanin even greater than asserted by Stilling.

Those bacteria most readily stained by the methly-violet were most readily influenced by it. The practical value in surgery of pyoktanin has been asserted by Stilling, by Sée and Moran, by Patterson, and by numerous other surgeons, and there would seem to be no doubt of the value of the remedy.

Pyoktanin is the discovery of Prof. J. Stilling, of Strassburg University, and in its introduction to the scientific public he claimed for it a true but harmless therapeutical disinfectant, using this latter term in the sense of a product that not only arrests or suspends the life-phenomena of micro-organisms, but destroys their vitality completely, beyond the possibility of resuscitation. Its safe yet powerful influence on animal tissues and fluids in the living body led him to claim for this discovery

**Visit Nashville in September and mingle with your colleagues.**

1. That it is devoid of any injurious effect on the human or animal economy or organism.

2. Certainly and utterly destructive of all bacteria or other micro-organisms absorbing it.

3. Rapidly and completely diffusible in and permeating both healthy and diseased animal tissue or fluids, and readily penetrating the enveloping membranes and internal substance of all bacterial colonies harbored by them.

Its non-poisonous character was regarded as an essential and safe quality for proper disinfection of all purulent, gangrenous, septic, syphilitic, tubercular, or other micro-parasitic morbid processes.

Of the other strong antiseptics or disinfectants, their dangerous toxic character when used of certain strengths was well known; while in mild dilution they were not of sufficient power to be reliable or efficacious in the destruction of micro-organisms. Others were prone to form insoluble compounds with portions of organic matter, thus serving as protecting coverings over strata of bacterial colonies which were somewhat removed from the surface and thorough sterilization of the inner layers of a diseased part not attained.

Its use was strongly urged in its first introduction in the treatment of corneal ulcers and suppurative conditions of the eye and its membranes—burns, purulent sores, and lacerations of various kinds. In epizootic eczema of horses and cattle it was found to be a rapid agent in their control, and moist eczemas were quickly amenable to its action.

Its value over corrosive sublimate, carbolic acid, iodoform, nitrate of silver, creosote, and chlorides, iodides, mercuric and metallic compounds, and phenols was highly extolled, but much of this has not been realized, because other remedies equally less dangerous than many of those quoted, over which pyoktanin was claimed to excel, have latterly been brought forth and have supplanted it in many directions. One very great objection to its use in powder form was the peculiar discoloration it produced, and which left on the hands of physician and patient such annoying and objectionable evidences of its application.

**Go down to Nashville and meet new faces.**

This coloring of blue or yellow, representing the two principal kinds used, was of some value in external applications as a guide to the operator's eye and hand, as to surface and depth covered or penetrated. The yellow is less objectionable than the blue, as regards coloring. Its use was advocated in pencil form, the yellow, the milder of the two, in the diseases of the eye, and the blue, the stronger, for ulcers, burns, or purulent wounds, the pencils to be dipped in water and the surfaces passed over until the parts take on the color of the pencil used. This plan was most useful in veterinary practice.

The pure pyoktanin powder to be strewn over purulent surfaces and then abandoned to spontaneous desquamation. A 2 per cent.

powder in moist eczemas or abrasions of the skin sprinkled over the parts. A $\frac{1}{10}$ per cent. powder in ophthalmic lesions. A 2 to 10 per cent. ointment in all conditions where this form would serve better than a powder. Solutions in water of the strength of 1 : 1000 to 1 : 100 as the antiseptic requirements indicate. These have been used for disinfecting purposes in rooms and of utensils used in connection with the sick, and in hospitals, prisons, and asylums. A $\frac{1}{10}$ per cent. dressing material has been on the market also—to be used in bandaging wounds or packing cavities.

Personally I have tried pyoktanin in a numler of cases; but I cannot say with any gratifying results, though it is equally true that in the cases where I used it every other remedy was equally unavailing. I used both the powder and pencil form, and gave it a very thorough trial in one case of summer sores or epitheliomatous condition of the skin. This mare had for the preceding two summers broke out in these never-healing patches, the prior season having been under my care, and the wounds did not heal properly until the cold weather. On the occurrence of the sores the third season I early resorted to the use of pyoktanin blue, in pencil form, and kept steadily at it until I was convinced that it was no better than the other remedies that had failed. On one large patch at the coronet I used it in powder form, and applied a pad of oakum with pressure-bandage, but this failed to give any better result than the pencil on the body, and finally when cold weather arrived they healed up, leaving rather unsightly patches over the body. This summer she did not break out for me, as I finally convinced the owner last winter of the wisdom of selling her, which he did.

**You will meet there some of your college classmates, on Sept. 7th, 8th, and 9th.**

I have used pyoktanin powder in fistulous withers, but not with any marked results that would lead me to favor its use over other remedies that have not the objectionable features of discoloring everything it touches and leaving its mark behind it, just as iodoform scents an entire stable or quarters where it is used.

Dr. H. D. Gill, of New York, has used it in purulent wounds and in punctured foot-wounds, and has had very good results with it.

Dr. Wilfred Lellman, of New York City, used it for several years very extensively in surgical practice for punctured or suppurating wounds, and commends it very highly in all such cases.

Dr. William H. Ridge reports a case of summer wound in a bay gelding. Location of sore on flank had been treated, and steadily became worse, until it spread to about three inches in diameter—very fetid. Pyoktanin in pencil form was used once daily without washing or any other application. In three days the wound was under control, and could not have healed more kindly. A second case of the same nature had been treated by the owner until it had spread to the size of a dollar. After ineffectually trying other remedies he began with pyoktanin, and it dried up in three days, and remained good for a week, when the owner thought he could handle it, but in two weeks it was as bad as ever.

**Renew old acquaintances at Nashville, Sept. 7th, 8th, and 9th.**

He reports its use in several cases of ordinary ulcers which healed nicely. Another case of wound on neck in front of withers, which began to have an offensive odor, had been treated with bluestone, white lotion, creolin, acetanilid, and boric acid, when a few applications of pyoktanin acted more favorably than any of the others. This animal was put to work before healing was complete.

Dr. A. T. Peters, of Nebraska, writes: "I am a great admirer of this remedy, as I have seen it used to a great extent in the Royal College of Stuttgart, and have used it very freely wherever I could. I have used it considerably in fistulous withers and in puncture of the coronet."

Dr. William Jakeman writes: "My first experience with it was in open-joints. In June, 1894, I was called up by telephone from a neighboring town forty miles distant. The gentleman informed me that his mare a week previously had run away and came in contact with a barbed-wire fence, laying her knee open two-thirds around. He had it sewed up and dressed by a local man, but, owing to the mare being a highly nervous animal, she acted badly and tore out the stitches. He could not get it to heal, and wanted to know if I advised him to shoot her. She was very well bred, and I recommended her being put on the train in my care. I have seen many cases of open joint, but never anything like that. The carpal bones could easily be felt. I had her placed in a sling with a nice-fitting splint made of sole leather to extend from foot to elbow and carefully secured with bandages, using compresses of tow under and above the knee to crowd the wound together. I kept the parts clean, and all the usual dressings were applied, but it did not do as well as I wished, and seeing pyoktanin spoken of in one of the journals—thanks to them for many points—I decided to get some and try it. I commenced to use it in the proportion of one drachm in a pint of water. With its use the discharge of synovia grew less, and the wound contracted and healed rapidly. I have used it in several cases of open joints since with equal success. I sometimes use it in conjunction with oleum caryophyllin, which I find to work nicely. I now use it 1 : 8 with petrolatum, and find it one of the best ointments for general use. Last winter a gentleman was driving a four-horse team when a horse from a grocery team ran away, coming in contact with the leaders, the shaft entering the breast and passing under the scapula and glanced along to the seventh rib. This proved very troublesome to heal, but I used pyoktanin, when it healed rapidly."

